# Serodiagnosis of Tuberculosis in Asian Elephants (*Elephas maximus*) in Southern India: A Latent Class Analysis

**DOI:** 10.1371/journal.pone.0049548

**Published:** 2012-11-16

**Authors:** Shalu Verma-Kumar, David Abraham, Nandini Dendukuri, Jacob Varghese Cheeran, Raman Sukumar, Kithiganahalli Narayanaswamy Balaji

**Affiliations:** 1 Department of Microbiology and Cell Biology, Indian Institute of Science, Bangalore, Karnataka, India; 2 Centre for Ecological Sciences, Indian Institute of Science, Bangalore, Karnataka, India; 3 Asian Nature Conservation Foundation, Bangalore, Karnataka, India; 4 Department of Medicine, McGill University, Montreal, Quebec, Canada; 5 Department of Epidemiology, Biostatistics and Occupational Health, McGill University, Montreal, Quebec, Canada; 6 Cheerans Lab (P) Limited, Thrissur, Kerala, India; Institut National de la Santé et de la Recherche Médicale U 872, France

## Abstract

**Background:**

*Mycobacterium tuberculosis*, a causative agent of chronic tuberculosis disease, is widespread among some animal species too. There is paucity of information on the distribution, prevalence and true disease status of tuberculosis in Asian elephants (*Elephas maximus*). The aim of this study was to estimate the sensitivity and specificity of serological tests to diagnose *M. tuberculosis* infection in captive elephants in southern India while simultaneously estimating sero-prevalence.

**Methodology/Principal Findings:**

Health assessment of 600 elephants was carried out and their sera screened with a commercially available rapid serum test. Trunk wash culture of select rapid serum test positive animals yielded no animal positive for *M. tuberculosis* isolation. Under Indian field conditions where the true disease status is unknown, we used a latent class model to estimate the diagnostic characteristics of an existing (rapid serum test) and new (four in-house ELISA) tests. One hundred and seventy nine sera were randomly selected for screening in the five tests. Diagnostic sensitivities of the four ELISAs were 91.3–97.6% (95% Credible Interval (CI): 74.8–99.9) and diagnostic specificity were 89.6–98.5% (95% CI: 79.4–99.9) based on the model we assumed. We estimate that 53.6% (95% CI: 44.6–62.8) of the samples tested were free from infection with *M. tuberculosis* and 15.9% (97.5% CI: 9.8 - to 24.0) tested positive on all five tests.

**Conclusions/Significance:**

Our results provide evidence for high prevalence of asymptomatic *M. tuberculosis* infection in Asian elephants in a captive Indian setting. Further validation of these tests would be important in formulating area-specific effective surveillance and control measures.

## Introduction

Conservation medicine enables us to rethink the linkages between human, animal, and environmental health [1], [2]. As wildlife populations become more fragmented and less genetically diverse stochastic events leading to disease outbreaks could become more common. A case in hand is that of the Asian elephant (*Elephas maximus*) an “Endangered” flagship species and featuring on the ‘2010 IUCN Red List of Threatened Species’ [3]. About 39,463-47,427 wild elephants are found in their 13 range countries in Asia [4]. Estimates of Asian elephant numbers in the wild in India are 26,000–28,000 of which 14,000 are found in southern India [5]. About 3467–3667 elephants are also held in captivity in India (http://envfor.nic.in/pe/PE%20Note.pdf) at forest camps, temples, zoological gardens and circuses, thus constituting a substantial population living in close proximity to humans.

Both humans and elephants are susceptible to infection primarily with *M. tuberculosis*. Importantly, TB is a serious zoonotic disease in elephants [6], [7], [8], [9], [10] and infects about 11-25% of tested captive elephant populations in USA, India and Nepal [11]. The transmission, pathobiology and immune correlates of TB are poorly understood in Asian elephants. There is paucity of information on the time intervals between exposure, seroconversion, and shedding of the bacilli as also latent versus active disease status. Equivalent to the culture of human sputum [12], [13], [14] trunk wash culture for isolation of *M. tuberculosis* remains the ‘gold standard’ of ante-mortem TB diagnostics in elephants [15]. In its absence, ante-mortem TB diagnosis presents a conundrum [16]. Clinical signs such as chronic weight loss, weakness, anorexia, exercise intolerance and abnormal discharge from the trunk [17] are frequently absent or seen at the terminal stages. Intradermal tuberculin test [17], [18] and radiographic thoracic evaluation [17] are unsuccessful in elephants. The GenProbe Amplified *Mycobacterium tuberculosis* Direct Test (MTD; Gen-Probe, San Diego, CA, USA) has found limited use [19], while ELISA [17], [20], restriction fragment length polymorphism (RFLP) [6], [17], [18] and serological tests such as the dual path platform (VetTB test), multi-antigen print immunoassay (MAPIA) and the rapid serum test (RT, ElephantTB STAT-PAK) from Chembio Diagnostics (Medford, USA) have also been evaluated in elephants [21], [22], [23].

In this study, we report screening of elephants with RT and four in-house ELISAs using *M. tuberculosis* H37Rv antigens *EsxA*-6 kDa early secretory antigenic target (ESAT-6) (Rv3875); *EsxB*-10 kDa culture filtrate antigen (CFP10) (Rv3874); PE_PGRS17 (Rv0978c) and PE_PGRS11 (Rv0754). The ESAT-6 and CFP10 proteins function in inducing interferon gamma (IFNγ) from memory effector cells upon infection with pathogenic mycobacteria [24], [25]. The proline glutamic acid (PE) and proline-proline-glutamic acid (PPE) families of acidic, glycine-rich proteins are unique to the Mycobacteria [26] and many function as cell surface antigens. The PE_PGRS11 (Rv0754) is a hypoxia responsive gene that encodes a functional phosphoglycerate mutase [27] and PE_PGRS17 and PE_PGRS11 antigens induce maturation and activation of human dendritic cells [28].

Performance characteristics of a diagnostic test should ideally enable us to distinguish between infected and non-infected animals. Notably, estimation of DSe (the proportion of infected animals correctly identified by the diagnostic test) and DSp (the proportion of non-infected animals accurately identified by the diagnostic test) of any index test is generally derived by comparing it to a standardized and validated ‘gold standard’ with the assumed sensitivity and specificity of 100% [29], [30], [31]. However, the gold standard could suffer from serious deficiencies. For example, the ante-mortem trunk wash culture for *M. tuberculosis*/*M. bovis* in Asian elephants is reported to suffer from poor sensitivity [17], [22], logistical issues in sample collection and processing and slow turnaround time. In view of these observations, we have utilized Latent Class Analysis (LCA) of five imperfect serological tests to estimate and derive the probability of *M. tuberculosis* infection in elephants.

## Results

### Sampling in Elephants

About 600 captive Asian elephants were visited for health assessment in the three southern Indian states of Kerala, Karnataka and Tamil Nadu. The sampling included healthy individuals as well as animals with alternative diagnosis such as chronic arthritis, impaction, and other non-specific symptoms such as anemia and emaciation. Sera from 179 animals were randomly selected for this study. Trunk-wash culture for isolation of *M. tuberculosis* in select RT positive elephants was carried out with no elephant testing positive. Post-mortem examination of one RT positive elephant revealed lung nodules from which *M. tuberculosis* was cultured. Ante-mortem serum from this animal was used as positive control in standardizing the ELISAs and immunoblot assay.

### Evaluation of Humoral Immunoreactivity

**Figure 1:**
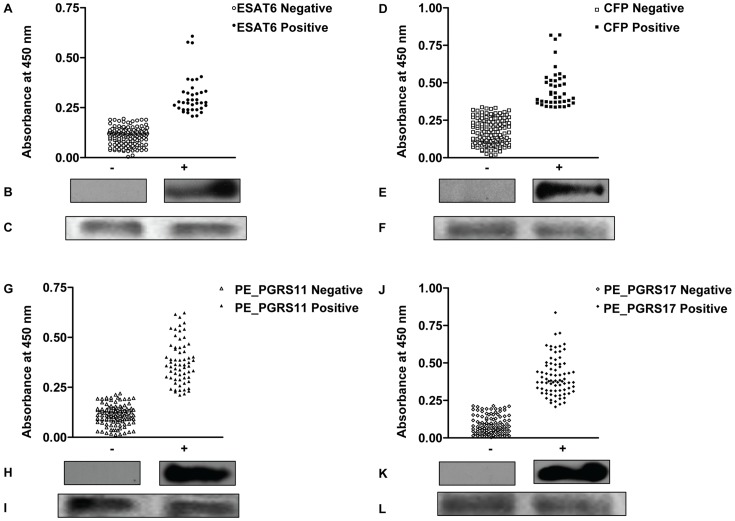
Differential humoral reactivity of four mycobacterial antigens with Asian elephant sera. For ELISA, elephant sera (1∶200) was allowed to react with ESAT-6 (1 µg/ml) (A), CFP10 (0.5 µg/ml) (D), PE_PGRS11 (0.25 µg/ml) (G) and PE_PGRS17 (0.25 µg/ml) (J). Scatter plots show the total number of animals testing seronegative and seropositive for each antigen. For immunoblotting, 10 µg of each transferred protein ESAT-6 (C), CFP10 (F), PE_PGRS11 (I) and PE_PGRS17 (L) was first stained with Ponceau to check for loading control. Next, individual lanes were cut out of the blot and probed with sera from reference negative and positive animals. B, E, H, K represent immunoblots for one representative negative and positive animal each. The westerns were not quantitative in nature.

One hundred and seventy nine elephants were screened for differential B-cell responses using RT and recombinant *M. tuberculosis* H37Rv antigens in ELISA format. Only selected reference samples were tested by immunoblot analysis using the four recombinant antigens and the results were not included in the LCA model. The immunoblots (Figure 1) were not quantitative in nature. The RT readout gave either a positive or negative test result (binary outcome), while the ELISA results were continuous numerical outcome values (continuous outcome). We dichotomized each continuous test result using a Weibull mixture model that assumed the elephants were a mixture of two latent groups – those with the antigen and those without. The cut-off value was the point of intersection of the two Weibull distributions. The cut-off values were 0.2 for ESAT-6, 0.337 for CFP10 and 0.22 for both PE_PGRS11 and PE_PGRS17. Test value greater than each cut-off was deemed to be a positive test. Of the 179 elephants 33 tested positive in RT, 37 in ESAT-6 ELISA, 41 in CFP10 ELISA, 64 in PE_PGRS11 ELISA and 78 in PE_PGRS17 ELISA (Figure 1).

### Analyzing Diagnostic Test Results using LCA

**Figure 2:**
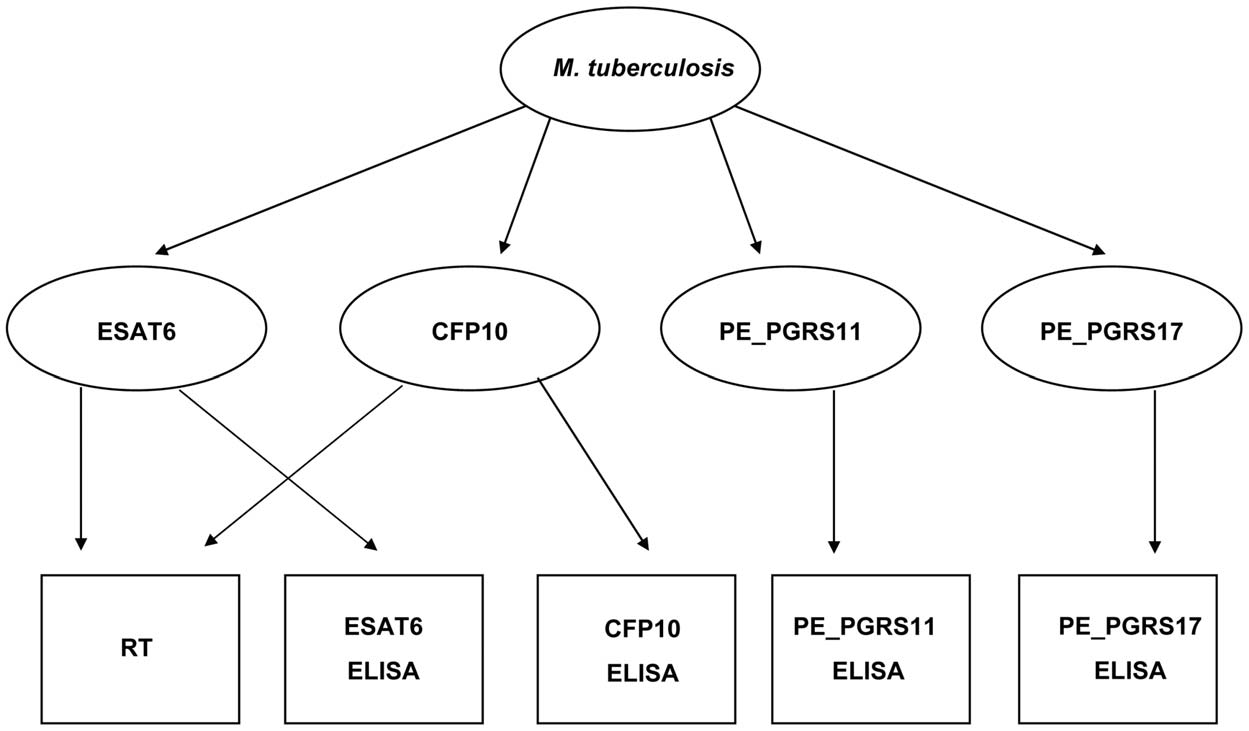
Schematic for the multiple latent variable model used in this study. The parameters to be estimated are depicted in oval shapes and the observed diagnostic test results are represented in rectangular shapes. While RT contains both the ESAT-6 and CFP10 antigens, the ELISAs are specific for one antigen each.

The LCA assumed in Figure 2 has sixteen latent classes. The sero-prevalence associated with each latent class is listed in Table 1. In the absence of any prior information, we chose to use a ‘non-informative’ prior distribution for the prevalence of *M. tuberculosis* infection, allowing for equal weight of all values from 0% to 100%. Using the posterior distribution, we report that 53.6% (97.5% CI: 44.6% to 62.8%) of the sera samples we tested did not carry any of the *M. tuberculosis* antibodies measured by the 5 tests. This estimate is higher than the 15% sero-prevalence reported by Abraham *et al.* (2008, Report submitted to Project Elephant, Ministry of Environment and Forests, Government of India) using RT in the same population of captive elephants. We report that the percentage of *M. tuberculosis* infected animals testing positive in all five tests is 15.9% (97.5% CI: 9.8%−24.0%). The DSe and DSp of each test with respect to the target antibody that it is designed to detect is listed in Table 2. Thus the PE_PGRS11 ELISA had the highest DSe of 97.6% (97.5% CI: 88.6%−99.8%) and DSp of 98.5% (97.5% CI: 93.6%−99.9%). Table 2 also lists the DSe and DSp of each test in detecting the presence of at least one antibody (Table 1 lists 15 latent classes that are positive for at least one antibody) as well as the latent antibodies it is not designed to detect. The main difference between our model and other latent class models is that we recognize that the different tests are measuring different latent variables. Thus, for example, we are able to comment not only on how well RT measures the antigens it is supposed to detect but also on how well it measures *M. tuberculosis* infection that is picked up by other antigens. Finally, the observed and predicted numbers of elephants for each combination of test results (Table 3) agree quite well suggesting that the model fits the data adequately.

**Table 1:** Defining the sixteen latent classes and calculating their sero-prevalence rates.

Class No.	Latent class definition	Seroprevalence	
		Median (95% CI)*	Mean
Class 1	Mtb**+, All antibodies+	15.9 (9.8, 24.0)	16.2
Class 2	Mtb+, ESAT6+, CFP10+, PE-PGRS11+	1.3 (0.1, 4.3)	1.5
Class 3	Mtb+, ESAT6+, CFP10+, PE-PGRS17+	0.4 (0.0, 2.1)	0.6
Class 4	Mtb+, ESAT6+, PE-PGRS11+, PE-PGRS17+	0.8 (0.0, 4.4)	1.2
Class 5	Mtb+, CFP10+, PE-PGRS11+, PE-PGRS17+	3.1 (0.2, 10.3)	3.7
Class 6	Mtb+, ESAT6+, CFP10+	0.8 (0.0, 3.2)	1.0
Class 7	Mtb+, ESAT6+, PE-PGRS11+	0.6 (0.0, 3.2)	0.9
Class 8	Mtb+, ESAT6+, PE-PGRS17+	0.5 (0.0, 2.3)	0.7
Class 9	Mtb+, CFP10+, PE-PGRS11+	0.5 (0.0, 2.6)	0.7
Class 10	Mtb+, CFP10+, PE-PGRS17+	0.4 (0.0, 2.3)	0.6
Class 11	Mtb+, PE-PGRS11+, PE-PGRS17+	11.9 (4.2, 18.5)	11.7
Class 12	Mtb+, ESAT6+	0.7 (0.0, 3.5)	1.0
Class 13	Mtb+, CFP10+	0.5 (0.0, 2.6)	0.7
Class 14	Mtb+, PE-PGRS11+	0.7 (0.0, 3.1)	0.9
Class 15	Mtb+, PE-PGRS17+	4.8 (0.2, 11.8)	5.1
Class 16	Mtb-, All antibodies-	53.6 (44.6, 62.8)	53.6

Latent class definition and the seroprevalence rate for each class thus defined in the LCA model for studying TB in Asian elephants in southern India. The latent classes are different combinations of the latent variables (i.e. true presence of the antibodies) which are present if *M. tuberculosis* infection is present. *Median estimate is at the 50% quantile while the 2.5% and 97.5% quantiles define a 95% credible interval (CI). **M. tuberculosis infection.

**Table 2:** Sensitivity (DSe) and specificity (DSp) of the five serological tests used in the study.

Test	w.r.t.**	DSe	DSp
		Median (95% CI)*	Median (95% CI)*
RT	At least 1 antibody	48.6 (37.2, 61.0)	99.3 (96.7, 99.9)
	ESAT6	44.6 (33.9, 56.4)	95.2 (90.4, 98.2)
	CFP10	46.5 (36.0, 58.1)	93.0 (87.8, 96.6)
	PE_PGRS11	79.8 (66.7, 90.8)	98.5 (93.6, 99.9)
	PE_PGRS17	84.5 (75.2, 91.3)	89.6 (79.4, 98.4)
ESAT6	At least 1 antibody	88.8 (73.5, 97.5)	95.1 (90.5, 98.2)
	ESAT6	91.3 (74.8, 99.5)	95.2 (90.4, 98.2)
	CFP10	77.0 (61.9, 88.5)	88.9 (83.2, 93.6)
	PE_PGRS11	85.0 (71.7, 94.0)	75.9 (68.2, 82.3)
	PE_PGRS17	80.3 (66.7, 90.2)	65.7 (57.8, 73.1)
CFP10	At least 1 antibody	88.8 (73.5, 97.5)	95.3 (90.8, 98.2)
	ESAT6	75.2 (60.0, 87.1)	91.2 (85.7, 95.2)
	CFP10	92.3 (76.9, 99.5)	93.0 (87.8, 96.6)
	PE_PGRS11	85.2 (71.5, 94.0)	76.0 (68.5, 82.6)
	PE_PGRS17	81.5 (67.9, 91.4)	66.2 (58.3, 73.5)
PE_PGRS11	At least 1 antibody	50.1 (38.2, 62.1)	93.9 (88.8, 97.2)
	ESAT6	47.1 (35.8, 58.7)	91.5 (85.9, 95.4)
	CFP10	49.1 (38.0, 60.2)	89.4 (83.5, 93.7)
	PE_PGRS11	97.6 (88.6, 99.8)	98.5 (93.6, 99.9)
	PE_PGRS17	87.1 (77.6, 93.6)	81.4 (73.4, 88.1)
PE_PGRS17	At least 1 antibody	44.4 (32.5, 57.6)	91.6 (85.5, 96.0)
	ESAT6	42.2 (30.9, 54.8)	89.6 (83.0, 94.1)
	CFP10	44.8 (33.6, 57.1)	87.8 (81.5, 92.7)
	PE_PGRS11	82.7 (68.2, 94.3)	91.9 (85.3, 96.5)
	PE_PGRS17	97.2 (88.5, 99.9)	89.6 (79.4, 98.7)

The LCA model was used to calculate the DSe and DSp of each test w.r.t. the antibody it is designed to detect as also presence of at least one antibody. *The Median estimate refers to the 50% quantile while the 2.5% and 97.5% quantiles define a 95% credible interval (CI). ** With respect to.

**Table 3:** Number of elephants (observed and predicted according to the latent class model) with each test result.

Binary Input	2.50%	50%	97.50%	Mean	Observed
00000	69	82	92	81	84
00001	9	16	26	17	16
00010	0	3	8	3	1
00011	18	24	29	24	26
00100	1	6	13	6	8
00101	0	1	4	1	1
00110	0	0	2	0	1
00111	0	2	6	2	0
01000	0	4	11	4	4
01001	0	1	3	1	1
01010	0	0	2	0	0
01011	0	1	5	2	2
01100	0	0	2	0	0
01101	0	0	1	0	0
01110	0	0	2	0	0
01111	0	2	8	3	2
10000	0	0	4	1	0
10001	0	0	1	0	0
10010	0	0	1	0	0
10011	0	1	3	1	1
10100	0	0	2	0	0
10101	0	0	2	0	0
10110	0	0	2	1	0
10111	1	4	8	4	4
11000	0	0	2	0	0
11001	0	0	2	0	0
11010	0	1	3	1	1
11011	0	3	8	3	2
11100	0	1	2	1	1
11101	0	0	3	1	0
11110	0	2	5	2	2
11111	12	19	25	19	22

Evaluating the fit of the model by comparing the observed and expected number of elephants with different combinations of tests to see if the assumptions of the substantive model in Figure 2 were satisfied. 50% refers to the median estimate while the 2.5% and 97.5% quantiles define a 95% credible interval (CI).

## Discussion

Latent class analysis is used in a scenario wherein the gold standard assessment of disease is unavailable and the true infection status unknown but the results of multiple imperfect tests are known [32], [33], [34]. This analysis is attractive because it does not arbitrarily treat one of the tests as a perfect gold-standard with 100% sensitivity and specificity. It allows intuitive construing of data for input and helps in understanding the uncertainties associated with the predicted prevalence estimates. A large number of reports using LCA in veterinary diagnostic tests have been published [35], [36], [37], [38], [39], [40]. This study uses LCA to estimate the diagnostic test characteristics of five serological tests and is the first report of this analysis used to study TB infection in elephants. This model recognizes that each test is measuring a different target latent variable, which is in turn associated with the presence of *M. tuberculosis* infection.

Test validation in the absence of suitable reference samples is extremely challenging [30], [31]. Culture for clinical isolation of *M. tuberculosis* remains the ‘gold standard’ of diagnostics; however a positive culture result is more likely in animals with advanced stage of disease. Validation of DSe based on positive culture results may result in its overestimation under field conditions [38], [40] and DSe may also vary with severity of disease. Validation of DSp entails testing of individuals and herds free from infection (a condition unlikely in TB endemic countries) [41] and may not be constant across different populations [38], [40]. Variables such as methods and their operational characteristics, expertise of the diagnostician, variations in host-pathogen interactions and difference in true disease prevalence rates may contribute to changes in performance of tests. Thus, test performance characteristics would need careful re-evaluation when used in different settings.

In order to decide the cut-off for our new in-house ELISAs, we had the choice of using either the RT as an imperfect gold standard or a mixture model. Even though the RT and ELISA work on the same biological principle, we did not use the former approach as the RT does not contain any PE_PGRS antigens. The mixture model for continuous data has been used for tuberculin skin test induration data among other applications [42]. The LCA is based on the premise that the true disease status is a common latent (or unobserved) variable associated with several imperfect tests that measure the same disease [43]. For the LCA, we delineated 16 latent classes which are the different combinations of the antibodies detected in the study. It would be interesting to link the latent classes to other disease parameters such as mortality and clinical signs. The disadvantage of using the LCA model is that it dichotomizes test results and thus does not use all the information derived from continuous test results. The choice of model used is dictated by the type of data and whether the model assumptions are satisfied. The model we propose is complex and subject to our interpretation. However, it incorporates results from five serological assays independent of culture results, estimates the true seroprevalence within the sampled population and the true disease status of each animal sampled therein.

Only about 10% of the *M. tuberculosis* proteome generates human antibody responses, and this immunoproteome contains predominantly membrane-associated and secreted proteins [44]. Differences in antibody profiles seen in TB patients need to factor in host characteristics, bacillary burden and metabolic state and protein expression by the infecting strain of *M. tuberculosis* [44], [45]. Additional complexity is introduced by the multiple clinical manifestations of disease in humans [46]. This host and pathogen derived heterogeneity has led to several reviews highlighting the shortcomings of TB immunodiagnostics [47], [48], [49], [50].

Circulating antibodies have been evaluated as biomarkers for TB since 1898 [51]. As compared to other members of the PE family (including PG_PGRS) or mycobacterial antigens, PE_PGR11 and PE_PGR17 have been shown to elicit stronger and differential antibody response in humans. Our laboratory has previously reported that PE_PGR11 and PE_PGR17 elicited antibodies in adult humans with active pulmonary infection, and in child patients with pulmonary or extrapulmonary TB [27], [28], [52]. Serology studies have demonstrated that antibodies reactive with a recombinant carboxyterminal fragment of the PE_PGRS protein from Rv1759c [53] or with the PGRS domain of Rv3367 [54] are present in human sera of patients infected with TB. The ESX protein family (for example, Rv3881c and Rv3784) are preferentially recognized antibody targets in active TB in humans [44]. The proteins CFP10 (Rv3784) and ESAT6 (Rv3785) have been evaluated in a number of veterinary serological assays [21], [22], [55], [56]. This approach is now giving way to whole proteome screening to identify TB associated proteins and the dynamics of antibody response they elicit during disease.

The past decade has seen decline of Asian elephant populations in most range countries with the exception of India and Sri Lanka [4]. Population depletion reduces the risk of host-specific infectious diseases except when the pathogen resides in reservoir hosts or when captivity results in increased infectious disease transmission [57]. Thus, conservation strategies that increase population density or cross-species contact such as in zoos, reserves and other captive conditions need careful evaluation in light of the risks of such infection. Cases of cross-species TB outbreaks in zoos are well documented [58]. The management of TB in Asian elephants in India remains inadequate owing to a number of issues including historical and cultural context of captivity of the species, legality of ownership (government owned vs. privately- owned) or the costs associated with the treatment. Albeit the United States Department of Agriculture has drafted clear guidelines [15] current need involves a creation of guidelines in regard to regulation of TB infections in elephants in India. Thus, our current study clearly proposes periodic verification/testing for TB in captive elephants as well as in-contact personnel.

Factors governing the initiation and expansion of ensuing immunity to wide ranging infections in elephants are not well understood. Further, detailed analysis of various effector roles played by the key components of immune systems such as T cells, B cells, macrophages or dendritic cells, complement and cytokines requires extensive investigation. For example, presence of five subclasses of IgG has been demonstrated in African elephant (*Loxodonta africana*) [59], [60]. Further, the genomic organization of the IgH, Igκ, and Igλ loci of the African elephant has been identified [61], [62]. Immunoreactivity analysis demonstrated the role for complement and antibodies during infections with African horse sickness [63], [64] Bluetongue [63] and Mycoplasmosis [65]. Interestingly, Alpha Napthyl Acetate Esterase activity was utilized as a T cell marker to demonstrate T lymphocyte distribution in peripheral blood [66] and presence of functional CD genes in the African elephant [67]. Not much tuberculosis disease stage-specific information is available in Asian elephants and vaccination with *M. bovis* BCG has not been evaluated in elephants. Lyaschenko *et al.* [21] reported that Multiantigen print immunoassay (MAPIA) and RT could pick up serum IgG to ESAT-6 and other proteins up to 3.5 years and 4.0 years respectively prior to culture of *M. tuberculosis* from trunk washes. They reported that ESAT6 and CFP10 were the immunodominant antigens elicited upon infection of elephants with *M. tuberculosis/M. bovis* [21], [23]. Significantly, in addition to ESAT-6 and CFP-10, we report high DSe and DSp for the two PE_PGRS ELISAs based on a latent class model that recognizes that the different tests are designed to measure different antigens. The DSe and DSp for the ELISAs we developed for ESAT6 and CFP10 were comparable to the commercially available RT test. Importantly, we have attempted to address immunoreactive potential as well as serodiagnostic utility of selected antigens of *M. tuberculosis* H37Rv which would help in our understanding of the pathophysiological attributes of TB infection in elephants.

Serology remains an attractive first step in TB diagnosis in wildlife. It is simple, quick, affordable and does not require repeated handling of animals. Once elicited, the antibody response is sustained while the trunk wash culture may yield intermittent results in elephants. Identifying serological correlates of active TB in elephants and their use in antitubercular treatment monitoring [21], [23], [68] could be potentially useful tools in situations where it is important to keep costs of diagnosis low. We are currently evaluating cell immunity based assays for TB diagnosis in elephants. Further studies into TB transmission and surveillance using accurate, low cost and high throughput assays are also warranted. Such active disease surveillance in elephant range countries would help us to study the dynamic relationship between TB and elephant conservation.

## Materials and Methods

### Study Population

In the three southern Indian states of Kerala, Karnataka and Tamil Nadu, there are an estimated 1,000 Asian elephants in captivity. These animals, mostly caught from the wild but also born in captivity, are maintained under different ownership and management regimens. A project for captive elephant health assessment was undertaken by Asian Nature Conservation Foundation (Permit No.8-1/2002-PE, Project Elephant, Ministry of Environment and Forests, Government of India). Apart from photographic documentation of body condition index, wounds and injuries with special reference to eyes and feet, routine haematology, serum biochemistry, urinalysis and dung analysis were performed for individual elephants. In an attempt to provide better healthcare to these elephants, the results of each elephant’s health evaluation was then handed over to the veterinarian in-charge of the elephant. Following a convenience/opportunity sampling method, nearly 600 elephants were visited over a period of one year. A random sample of 179 serum samples collected from this heath survey was selected for this study.

### Materials

Unless otherwise specified all materials used in this study were purchased from Sigma-Aldrich, St. Louis, USA. Vacutainer needles (No. 301747), Vacutainer tubes (No. 367820), PANTA™ Supplement, BACTEC™ 12B Mycobacteria Culture Vials and BBL™ Mycobactose Lowenstein-Jensen Medium were procured from BD (Franklin Lakes, NJ, USA). GammaBind G, Type 2 affinity matrix was bought from GE Healthcare Bio-Sciences (Uppsala, Sweden), Ni-nitrilotriacetic acid (Ni-NTA) columns from Qiagen (Valencia, CA, USA), Nunc-Immuno Plates (No 44204) from NUNC A/S (Roskilde, Denmark) and polyvinylidene difluoride membranes (PVDF) from Millipore (Bedford, MA, USA). ECL detection system was bought from Perkin-Elmer (MA, USA) and 3,3′,5,5′-tetramethylbenzidine (TMB) and horse radish peroxidase (HRP)-labeling kit from Bangalore Genei (Bangalore, Karnataka, India).

### Collection of Serum and Screening with RT

Blood was collected by venipuncture of the middle auricular vein, allowed to clot at room temperature and serum separated within three hours of collection by centrifugation at 500 *g* for 10 minutes. Each serum was screened with RT and stored at −70°C until further testing. The RT (http://www.chembio.com/animaltest4.html) is a point-of-care lateral flow serological test licensed by the USDA in 2007. Greenwald *et al.* [22] reported a DSp of 95.2% (95% CI, 90.1 to 97.9) and DSe of 100% (95% CI, 84.0%−100%) for the RT.

### Trunk-wash Culture for Isolation of *M. tuberculosis*

The procedure for trunk wash collection as described in the Guidelines for the Control of Tuberculosis in Elephants, 2008 [15] was modified [69]. Trunk wash specimens from select RT positive elephants were tested for *M. tuberculosis* culture [15]. Briefly, 0.5 ml of sample supplemented with Erythromysin (32 µg/ml) and PANTA™ Supplement was inoculated into BACTEC™ 12B vials and BBL™ Mycobactose Lowenstein-Jensen Medium and grown at 37°C and 10% CO_2_ for 8 weeks; this was followed by a niacin-nitrate reduction test for confirming *M. tuberculosis*. Ante-mortem serum collected from one elephant, which showed nodules in the lung tissue during post-mortem examination and from which *M. tuberculosis* was cultured on Lowenstein-Jensen medium, was used as positive control in the serological assays.

### Expression and Purification of Recombinant ESAT6, CFP10, PE-PGRS17 AND PE-PGRS11 Proteins

*M. tuberculosis* H37Rv antigens *EsxA*-6 kDa early secretory antigenic target (ESAT-6) (Rv3875); *EsxB*-10 kDa culture filtrate antigen (CFP10) (Rv3874); PE_PGRS17 (Rv0978c) and PE_PGRS11 (Rv0754) were used as the major antigenic determinants in this study. Recombinant expression vectors for Rv3875, Rv3874, Rv0978c and Rv0754 antigens were obtained from Colorado State University, TB Vaccine Testing and Research Materials Contract (http://www.cvmbs.colostate.edu/mip/tb/recombinant.htm) and expressed as described previously [70]. Briefly, for the expression of His-tagged recombinant antigens, *Escherichia coli* BL21(DE3) cells carrying recombinant plasmids were induced with isopropyl-b-D-thiogalactopyranoside, and the proteins purified under native conditions (ESAT6 and CFP10) and denaturing conditions (PE_PGRS17 and PE_PGRS11) using Ni-NTA columns. In-gel digestions of proteins for matrix-assisted laser desorption/ionization mass spectrometry was carried out for identification.

### Raising Rabbit Anti-Asian Elephant IgG-horse Radish Peroxidase (HRP)

Asian elephant IgG was separated from sera [71] and rabbit anti-Asian elephant IgG raised as per [59] and [60] with modifications. Briefly, New Zealand white rabbits were injected subcutaneously at multiple sites with 1 mg of purified Asian elephant IgG emulsified in equal volume of Freund’s complete adjuvant followed by a second dose of 500 µg Asian elephant IgG emulsified in Freund’s incomplete adjuvant. Antibody titres in sera were determined two weeks post final immunization by ELISA. The rabbit anti-Asian elephant IgG was coupled to HRP and its reactivity to elephant IgG was checked. All animal experiments were approved by the Institutional Ethics Committee for Animal Experimentation and Institutional Biosafety Committee, Indian Institute of Science, Bangalore.

### ELISA

Careful checker board titration for optimum protein concentration, elephant sera dilution, rabbit anti-Asian elephant IgG-HRP was carried out for each individual ELISA. All protein dilutions were made in 1X PBS (137 mM NaCl; 2.7 mM KCl; 4.3 mM Na2HPO4; 1.47 mM KH2PO4, pH 7.4). 1X PBST (1X PBS with 0.05% tween-20) was used as wash buffer and 3% BSA in PBST as blocking buffer. Elephant sera, rabbit anti-Asian elephant IgG-HRP were each diluted in blocking buffer and 100 µl added per well. ESAT-6 (1 µg/ml), CFP10 (0.5 µg/ml), PE_PGRS17 and PE_PGRS11 (0.25 µg/ml each) were coated overnight (o/n) at 4°C into ELISA plates and then washed thrice. Blocking for 1 hour was followed by addition of elephant sera (1∶200) and incubation o/n at 4°C. After washing, rabbit anti-Asian elephant IgG-HRP (1∶3000) was added and the plate incubated o/n at 4°C. Tetramethylbenzidine was used as chromogenic substrate and the absorbance was read at 450 nm using an ELISA reader (Molecular Devices, Sunnyvale, CA, USA).

### Immunoblot Analysis

Ten µg of purified protein was subjected to 12% SDS-PAGE (Laemmeli) or 10% Tricine SDS-PAGE; transferred to PVDF and stained with Ponceau to check for loading control. The PVDF was cut into strips, blocked with 5% nonfat dried milk and each strip probed with individual elephant sera overnight at 4°C, probed with rabbit anti-Asian elephant IgG-HRP and the blot visualized with the ECL detection system.

### Statistical Analysis

To adjust for the imperfect nature of the gold-standard reference, our test validation entailed i) determining an optimal cut-off for each ELISA using a mixture model for continuous data ii) using LCA to estimate the DSe and DSp of the five dichotomous tests. The first step was carried out using the *mixdist* library in the R software package [72] assuming that the observed continuous data on each test arises from a mixture of two Weibull distributions among the antibody positive and antibody negative elephants. The point of intersection of the two density functions was chosen as the optimal cut-off.

The LCA was carried out using the *lcmr* library in R software package [43]. This package uses a Bayesian approach to estimate the parameters of interest. We used non-informative prior distributions over all parameters so as to let the data dominate the analysis. The LCA model (Figure 2) assumed that each of the ELISA tests was measuring a different latent variable (i.e. true presence of the antibody) which was present if *M. tuberculosis* infection was present. Thus, this model adjusts for the correlation that may arise between tests within the groups of elephants that are *M. tuberculosis* infection positive or negative. The RT test was assumed to measure the presence of both ESAT-6 and CFP10 antibodies while each of the four ELISA was assumed to measure the presence of the corresponding antibody. The resulting model had 16 latent classes corresponding to different combinations of the antibodies (see supplementary document File S1). The fit of the model was evaluated by comparing the observed and expected number of elephants with different combinations of tests and a posterior predictive check for conditional dependence (Table 3). The predictive values of each test combination were examined to see if the assumptions of the substantive model in Figure 2 were satisfied.

## Supporting Information

File S1 **Algorithm for LCA model.**
(DOC)
