# Correction: Serodiagnosis of Tuberculosis in Asian Elephants (*Elephas maximus*) in Southern India: A Latent Class Analysis

**DOI:** 10.1371/annotation/fb3b82df-f9b7-4c6a-a952-2f86e5fe5f48

**Published:** 2012-12-18

**Authors:** Shalu Verma-Kumar, David Abraham, Nandini Dendukuri, Jacob Varghese Cheeran, Raman Sukumar, Kithiganahalli Narayanaswamy Balaji

Authors David Abraham and Kithiganahalli Narayanaswamy Balaji should not have been attributed equal contributions to the article. Shalu Verma-Kumar is the only first author.

