# Correction: Serodiagnosis of Tuberculosis in Asian Elephants (*Elephas maximus*) in Southern India: A Latent Class Analysis

**DOI:** 10.1371/journal.pone.0294550

**Published:** 2023-11-13

**Authors:** Shalu Verma-Kumar, David Abraham, Nandini Dendukuri, Jacob Varghese Cheeran, Raman Sukumar, Kithiganahalli Narayanaswamy Balaji

The ponceau loading control panels in Fig 1F and 1L of this article [1, corrected in 2] appear more similar than would be expected from independent experimental results. The original uncropped images corresponding to the results in this figure are no longer available, however replicate data is available.

**Fig 1:**
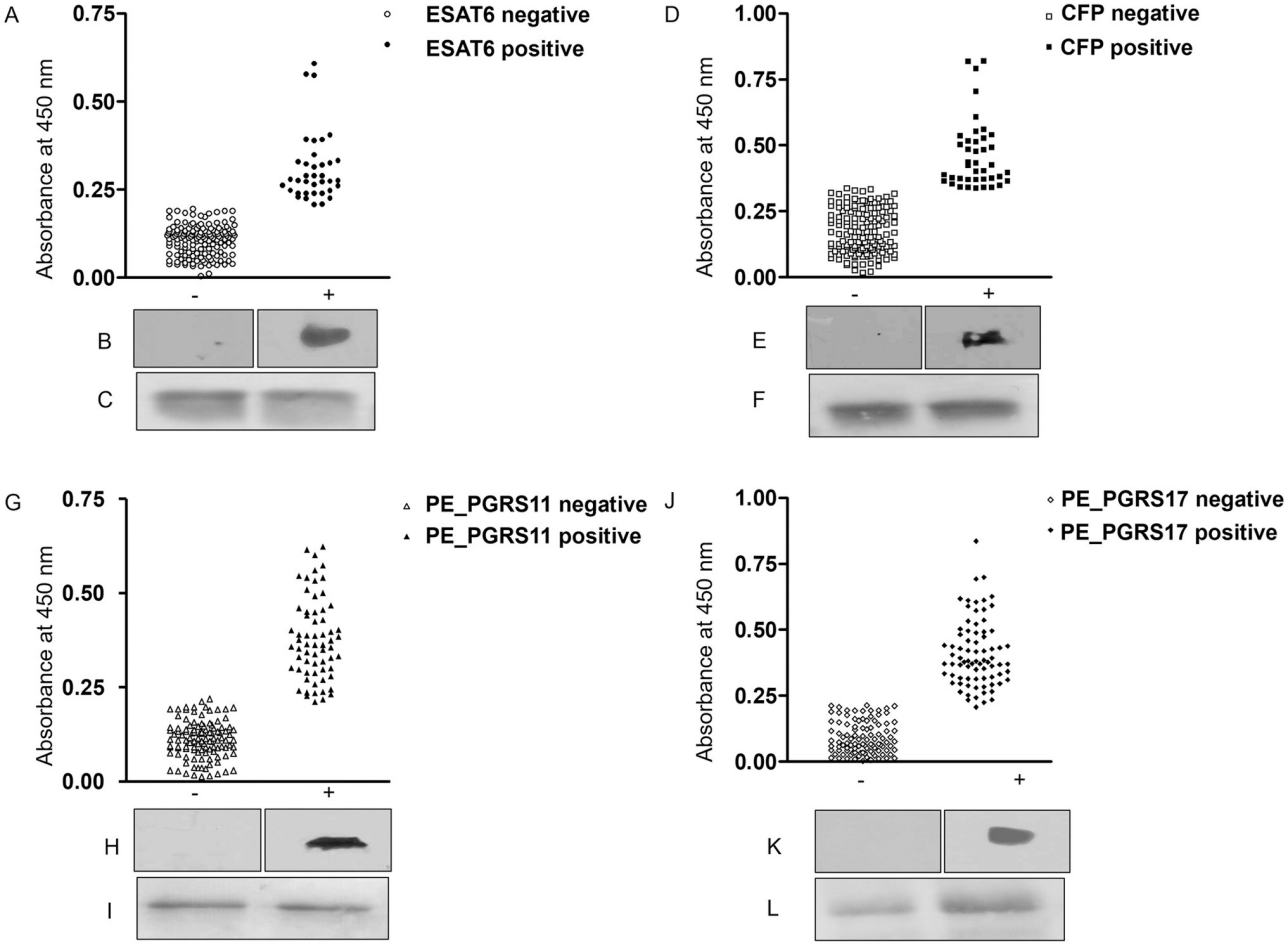
Differential humoral reactivity of four mycobacterial antigens with Asian elephant sera. For ELISA, elephant sera (1∶200) was allowed to react with ESAT-6 (1 μg/ml) (A), CFP10 (0.5 μg/ml) (D), PE_PGRS11 (0.25 μg/ml) (G) and PE_PGRS17 (0.25 μg/ml) (J). Scatter plots show the total number of animals testing seronegative and seropositive for each antigen. For immunoblotting, 10 μg of each transferred protein ESAT-6 (C), CFP10 (F), PE_PGRS11 (I) and PE_PGRS17 (L) was first stained with Ponceau to check for loading control. Next, individual lanes were cut out of the blot and probed with sera from reference negative and positive animals. B, E, H, K represent immunoblots for one representative negative and positive animal each. The westerns were not quantitative in nature.

The authors provide an updated version of Fig 1 with this notice containing the replicate image data for Fig 1B, 1C, 1E, 1F, 1H, 1I, 1K and 1L. Original uncropped images for Western blotting and ponceau staining are provided in S1 File, and quantitative data underlying the results in Figure 1A, 1D, 1J is provided in S2 File.

The raw data underlying Fig 1G and the summary results in Tables 1–3 are no longer available, however the original underlying data to support all other results in the article and Supporting Information files are available from the corresponding author.

The authors apologize for the error in the published article.

## Supporting information

S1 File Original uncropped Western blot and ponceau staining images underlying replicate data in the updated Fig 1. (PPTX)

S2 File Quantitative data underlying results in Fig 1A, 1D and 1J. (XLSX)
