# Supplementary material for: Serodiagnosis of Tuberculosis in Asian Elephants (Elephas maximus) in Southern India: A Latent Class Analysis
Source: PLoS One. 2012 Nov 16;7(11):e49548. doi: 10.1371/journal.pone.0049548 (PMC3500311; doi:10.1371/journal.pone.0049548)
Supplement: File S1 — Algorithm for LCA model. (DOC) [file pone.0049548.s001.doc]

**Supporting Information**

**S1. Algorithm for LCA model**

sample.shalu2<-read.table("N:\\Software\\lcmr\\shalutest\\Final_input.txt",header=T)

##---------- shalu.4.classes --------------------------

cstA <- matrix(0, 4, 5)

cstA[1,] <- seq(1 ,5) # Label: Antibody+, Pathogen+

cstA[2,] <- seq(6 ,10) # Label: Antibody+, Pathogen-

cstA[3,] <- seq(11 ,15) # Label: Antibody-, Pathogen+

cstA[4,] <- seq(16 ,20) # Label: Antibody-, Pathogen-

cstB <- matrix(0, 4, 5)

lcmr.obj <- lcmr(sample.shalu2, cstA, cstB)

init <- list(prev=c(0.1,0.1,0.1,0.7), a= c(rep(2, 10),rep(-2, 10)))

shalu.4.classes <- gs(lcmr.obj, iter=11000, init=init)

##---------- shalu.3.classes --------------------------

cstA <- matrix(0, 3, 5)

cstA[1,] <- seq(1 ,5) # Label: Antibody+, Pathogen+

cstA[2,] <- seq(6 ,10) # Label: Antibody+, Pathogen+

cstA[3,] <- seq(11 ,15) # Label: Antibody-, Pathogen-

cstB <- matrix(0, 3, 5)

lcmr.obj <- lcmr(sample.shalu2, cstA, cstB)

init <- list(prev=c(0.2,0.1, 0.7), a= c(rep(2, 5),rep(-2, 10)))

shalu.3.classes <- gs(lcmr.obj, iter=11000, init=init)

##---------- shalu.2.classes --------------------------

cstA <- matrix(0, 2, 5)

cstA[1,] <- seq(1 ,5) # Label: Antibody+, Pathogen+

cstA[2,] <- seq(6 ,10) # Label: Antibody-, Pathogen+

cstB <- matrix(0, 2, 5)

lcmr.obj <- lcmr(sample.shalu2, cstA, cstB)

init <- list(prev=c(0.8,0.7), a= c(rep(2, 5),rep(-2, 5)))

shalu.2.classes <- gs(lcmr.obj, iter=11000, init=init)

##---------- shalu.3.classes.constraints1 --------------------------

cstA <- matrix(0, 3, 5)

cstA[1,] <- seq(1 ,5) # Label: Antibody+, Pathogen+

cstA[2,] <- seq(6 ,10) # Label: Antibody+, Pathogen-

cstA[3,] <- seq(11 ,15) # Label: Antibody-, Pathogen-

cstB <- matrix(0, 3, 5)

lcmr.obj <- lcmr(sample.shalu2, cstA, cstB)

init <- list(prev=c(0.2,0.1, 0.7), a= c(rep(2, 5),rep(-2, 10)))

shalu.3.classes <- gs(lcmr.obj, iter=11000, init=init,prior.mA=c(5,rep(0,14)),prior.sdA=c(0.01,rep(1,14)))

##---------- shalu.4.classes.constraints1 --------------------------

cstA <- matrix(0, 4, 5)

cstA[1,] <- seq(1 ,5) # Label: Mtb+, All antigens+

cstA[2,] <- c(1,2,3,6,7) # Label: Mtb+, ESAT6 or CFP10+, PE-PGRS-

cstA[3,] <- c(8,9,10,4,5) # Label: Mtb+, ESAT6 or CFP10-, PE-PGRS+

cstA[4,] <- c(8,9,10,6,7) # Label: Mtb-, All antigens-

cstB <- matrix(0, 4, 5)

lcmr.obj <- lcmr(sample.shalu2, cstA, cstB)

init <- list(prev=c(0.1,0.1,0.1,0.7), a= c(rep(2, 5),rep(-2, 5)))

shalu.4.classes.constraint1 <- gs(lcmr.obj, iter=11000, init=init)

##---------- shalu.4.classes.constraints2 --------------------------

cstA <- matrix(0, 5, 5)

cstA[1,] <- seq(1 ,5) # Label: Mtb+, All antigens+

cstA[2,] <- c(1,2,3,6,7) # Label: Mtb+, ESAT6 or CFP10+, PE-PGRS-

cstA[3,] <- c(8,9,10,4,5) # Label: Mtb+, ESAT6 or CFP10-, PE-PGRS+

cstA[4,] <- c(8,9,10,6,7) # Label: Mtb+, All antigens-

cstA[5,] <- seq(11,15) # Label: Mtb-, All antigens-

cstB <- matrix(0, 4, 5)

lcmr.obj <- lcmr(sample.shalu2, cstA, cstB)

init <- list(prev=c(0.1,0.1,0.1,0.7), a= c(rep(2, 5),rep(-2, 10)))

shalu.4.classes.constraint2 <- gs(lcmr.obj, iter=11000, init=init)

##---------- shalu.16.classes.constraint1 --------------------------

cstA <- matrix(0, 16, 5)

cstA[1,] <- seq(1 ,5) # Label: Mtb+, All antigens+

cstA[2,] <- c(1,2,3,4,10) # Label: Mtb+, ESAT6+, CFP10+, PE-PGRS11+

cstA[3,] <- c(1,2,3,9,5) # Label: Mtb+, ESAT6+, CFP10+, PE-PGRS17+

cstA[4,] <- c(1,2,8,4,5) # Label: Mtb+, ESAT6+, PE-PGRS11+, PE-PGRS17+

cstA[5,] <- c(1,7,3,4,5) # Label: Mtb+, CFP10+, PE-PGRS11+, PE-PGRS17+

cstA[6,] <- c(1,2,3,9,10) # Label: Mtb+, ESAT6+, CFP10+

cstA[7,] <- c(1,2,8,4,10) # Label: Mtb+, ESAT6+, PE-PGRS11+

cstA[8,] <- c(1,2,8,9,5) # Label: Mtb+, ESAT6+, PE-PGRS17+

cstA[9,] <- c(1,7,3,4,10) # Label: Mtb+, CFP10+, PE-PGRS11+

cstA[10,] <- c(1,7,3,9,5) # Label: Mtb+, CFP10+, PE-PGRS17+

cstA[11,] <- c(6,7,8,4,5) # Label: Mtb+, PE-PGRS11+, PE-PGRS17+

cstA[12,] <- c(1,2,8,9,10) # Label: Mtb+, ESAT6+

cstA[13,] <- c(1,7,3,9,10) # Label: Mtb+, CFP10+

cstA[14,] <- c(6,7,8,4,10) # Label: Mtb+, PE-PGRS11+

cstA[15,] <- c(6,7,8,9,5) # Label: Mtb+, PE-PGRS17+

cstA[16,] <- seq(6,10) # Label: Mtb-, All antigens-

cstB <- matrix(0, 16, 5)

lcmr.obj <- lcmr(sample.shalu2, cstA, cstB)

init <- list(prev=c(0.2,rep(0.2/14,14),0.6), a= c(rep(2, 5),rep(-2, 5)))

shalu.16.classes.constraint1 <- gs(lcmr.obj, iter=11000, init=init)

ss(shalu.16.classes.constraint1,1001,10000,D=seq(1,15))

ss(shalu.16.classes.constraint1,1001,10000,D=c(1,2,3,4,6,7,8,12))

ss(shalu.16.classes.constraint1,1001,10000,D=c(1,2,3,5,6,9,10,13))

ss(shalu.16.classes.constraint1,1001,10000,D=c(1,2,4,5,7,9,11,14))

ss(shalu.16.classes.constraint1,1001,10000,D=c(1,3,4,5,8,10,11,15))

##---------- shalu.17.classes.constraint1 --------------------------

cstA <- matrix(0, 17, 5)

cstA[1,] <- seq(1 ,5) # Label: Mtb+, All antigens+

cstA[2,] <- c(1,2,3,4,10) # Label: Mtb+, ESAT6+, CFP10+, PE-PGRS11+

cstA[3,] <- c(1,2,3,9,5) # Label: Mtb+, ESAT6+, CFP10+, PE-PGRS17+

cstA[4,] <- c(1,2,8,4,5) # Label: Mtb+, ESAT6+, PE-PGRS11+, PE-PGRS17+

cstA[5,] <- c(1,7,3,4,5) # Label: Mtb+, CFP10+, PE-PGRS11+, PE-PGRS17+

cstA[6,] <- c(1,2,3,9,10) # Label: Mtb+, ESAT6+, CFP10+

cstA[7,] <- c(1,2,8,4,10) # Label: Mtb+, ESAT6+, PE-PGRS11+

cstA[8,] <- c(1,2,8,9,5) # Label: Mtb+, ESAT6+, PE-PGRS17+

cstA[9,] <- c(1,7,3,4,10) # Label: Mtb+, CFP10+, PE-PGRS11+

cstA[10,] <- c(1,7,3,9,5) # Label: Mtb+, CFP10+, PE-PGRS17+

cstA[11,] <- c(6,7,8,4,5) # Label: Mtb+, PE-PGRS11+, PE-PGRS17+

cstA[12,] <- c(1,2,8,9,10) # Label: Mtb+, ESAT6+

cstA[13,] <- c(1,7,3,9,10) # Label: Mtb+, CFP10+

cstA[14,] <- c(6,7,8,4,10) # Label: Mtb+, PE-PGRS11+

cstA[15,] <- c(6,7,8,9,5) # Label: Mtb+, PE-PGRS17+

cstA[16,] <- seq(6,10) # Label: Mtb+, All antigens-

cstA[17,] <- seq(11,15) # Label: Mtb+, All antigens-

cstB <- matrix(0, 17, 5)

lcmr.obj <- lcmr(sample.shalu2, cstA, cstB)

init <- list(prev=c(0.2,rep(0.2/14,15),0.6), a= c(rep(2, 5),rep(0,5),rep(-2, 5)))

shalu.17.classes.constraint1 <- gs(lcmr.obj, iter=11000, init=init)

##---------- shalu.16.classes.constraint1+RE --------------------------

cstA <- matrix(0, 16, 5)

cstA[1,] <- seq(1 ,5) # Label: Mtb+, All antigens+

cstA[2,] <- c(1,2,3,4,10) # Label: Mtb+, ESAT6+, CFP10+, PE-PGRS11+

cstA[3,] <- c(1,2,3,9,5) # Label: Mtb+, ESAT6+, CFP10+, PE-PGRS17+

cstA[4,] <- c(1,2,8,4,5) # Label: Mtb+, ESAT6+, PE-PGRS11+, PE-PGRS17+

cstA[5,] <- c(1,7,3,4,5) # Label: Mtb+, CFP10+, PE-PGRS11+, PE-PGRS17+

cstA[6,] <- c(1,2,3,9,10) # Label: Mtb+, ESAT6+, CFP10+

cstA[7,] <- c(1,2,8,4,10) # Label: Mtb+, ESAT6+, PE-PGRS11+

cstA[8,] <- c(1,2,8,9,5) # Label: Mtb+, ESAT6+, PE-PGRS17+

cstA[9,] <- c(1,7,3,4,10) # Label: Mtb+, CFP10+, PE-PGRS11+

cstA[10,] <- c(1,7,3,9,5) # Label: Mtb+, CFP10+, PE-PGRS17+

cstA[11,] <- c(6,7,8,4,5) # Label: Mtb+, PE-PGRS11+, PE-PGRS17+

cstA[12,] <- c(1,2,8,9,10) # Label: Mtb+, ESAT6+

cstA[13,] <- c(1,7,3,9,10) # Label: Mtb+, CFP10+

cstA[14,] <- c(6,7,8,4,10) # Label: Mtb+, PE-PGRS11+

cstA[15,] <- c(6,7,8,9,5) # Label: Mtb+, PE-PGRS17+

cstA[16,] <- seq(6,10) # Label: Mtb-, All antigens-

cstB1 <- matrix(0, 16, 5)

cstB1[5,] <-c(1,0,0,2,3)

cstB=list(cstB1=cstB1)

lcmr.obj <- lcmr(sample.shalu2, cstA, cstB)

init <- list(prev=c(0.2,rep(0.2/14,14),0.6), a= c(rep(2, 5),rep(-2, 5)),b=rep(0.5,3))

shalu.16.classes.constraint1.re <- gs(lcmr.obj, iter=11000, init=init)

ss(shalu.16.classes.constraint1.re,1,5000,D=seq(1,15))

ss(shalu.16.classes.constraint1.re,1,5000,D=c(1,2,3,4,6,7,8,12))

ss(shalu.16.classes.constraint1.re,1,5000,D=c(1,2,3,5,6,9,10,13))

ss(shalu.16.classes.constraint1.re,1,5000,D=c(1,2,4,5,7,9,11,14))

ss(shalu.16.classes.constraint1.re,1,5000,D=c(1,3,4,5,8,10,11,15))

##---------- shalu.2.classes --------------------------

cstA <- matrix(0, 2, 5)

cstA[1,] <- seq(1 ,5) # Label: Antibody+, Pathogen+

cstA[2,] <- seq(6 ,10) # Label: Antibody+, Pathogen-

cstB <- matrix(0, 2, 5)

lcmr.obj <- lcmr(sample.shalu2, cstA, cstB)

init <- list(prev=c(0.3,0.7), a= c(rep(2, 5),rep(-2, 5)))

shalu.2.classes <- gs(lcmr.obj, iter=11000, init=init)

##---------- shalu.2.classes+RE --------------------------

cstA <- matrix(0, 2, 5)

cstA[1,] <- seq(1 ,5) # Label: Antibody+, Pathogen+

cstA[2,] <- seq(6 ,10) # Label: Antibody+, Pathogen-

cstB1 <- matrix(0, 2, 5)

cstB2 <- matrix(0, 2, 5)

cstB2[2,]<-c(0,0,0,1,1)

cstB <- list(cstB1,cstB2)

lcmr.obj <- lcmr(sample.shalu2, cstA, cstB)

init <- list(prev=c(0.3,0.7), a= c(rep(2, 5),rep(-2, 5)),b=rep(1,1))

shalu.2.classes.re <- gs(lcmr.obj, iter=11000, init=init)

##---------- shalu.3.classes --------------------------

cstA <- matrix(0, 3, 5)

cstA[1,] <- seq(1 ,5) # Label: Antibody+, Pathogen+

cstA[2,] <- seq(6 ,10) # Label: Antibody+, Pathogen-

cstA[3,] <- seq(11 ,15) # Label: Antibody+, Pathogen-

cstB <- matrix(0, 3, 5)

lcmr.obj <- lcmr(sample.shalu2, cstA, cstB)

init <- list(prev=c(0.1,0.2,0.7), a= c(rep(2, 10),rep(-2, 5)))

shalu.3.classes <- gs(lcmr.obj, iter=11000, init=init)

##---------- shalu.3.classes --------------------------

cstA <- matrix(0, 3, 5)

cstA[1,] <- seq(1 ,5) # Label: Antibody+, Pathogen+

cstA[2,] <- seq(6 ,10) # Label: Antibody+, Pathogen-

cstA[3,] <- seq(11 ,15) # Label: Antibody+, Pathogen-

cstB <- matrix(0, 3, 5)

lcmr.obj <- lcmr(sample.shalu2, cstA, cstB)

init <- list(prev=c(0.1,0.2,0.7), a= c(rep(2, 10),rep(-2, 5)))

shalu.3.classes <- gs(lcmr.obj, iter=11000, init=init)

##---------- shalu.8.classes --------------------------

cstA <- matrix(0, 8, 5)

cstA[1,] <- seq(1 ,5) #Mtb+, ESAT6+, CFP10+, PE-PGRS+

cstA[2,] <- c(1,2,3,9,10) #Mtb+, ESAT6+, CFP10+, PE-PGRS-

cstA[3,] <- c(1,2,8,4,5) #Mtb+, ESAT6+, CFP10-, PE-PGRS+

cstA[4,] <- c(1,2,8,9,10) #Mtb+, ESAT6+, CFP10-, PE-PGRS-

cstA[5,] <- c(1,7,3,4,5) #Mtb+, ESAT6-, CFP10+, PE-PGRS+

cstA[6,] <- c(1,7,3,9,10) #Mtb+, ESAT6-, CFP10+, PE-PGRS-

cstA[7,] <- c(6,7,8,4,5) #Mtb+, ESAT6-, CFP10-, PE-PGRS+

cstA[8,] <- c(6,7,8,9,10) #Mtb+, ESAT6-, CFP10-, PE-PGRS-

cstB <- matrix(0, 8, 5)

lcmr.obj <- lcmr(sample.shalu2, cstA, cstB)

init <- list(prev=rep(1/8,8), a= c(rep(2, 5),rep(-2, 5)))

shalu.8.classes <- gs(lcmr.obj, iter=11000, init=init)
