# Supplementary figures and images for: Correction: Serodiagnosis of Tuberculosis in Asian Elephants (Elephas maximus) in Southern India: A Latent Class Analysis
Source: PLoS One. 2023 Nov 13;18(11):e0294550. doi: 10.1371/journal.pone.0294550 (PMC10642802; doi:10.1371/journal.pone.0294550)

## Slide 1
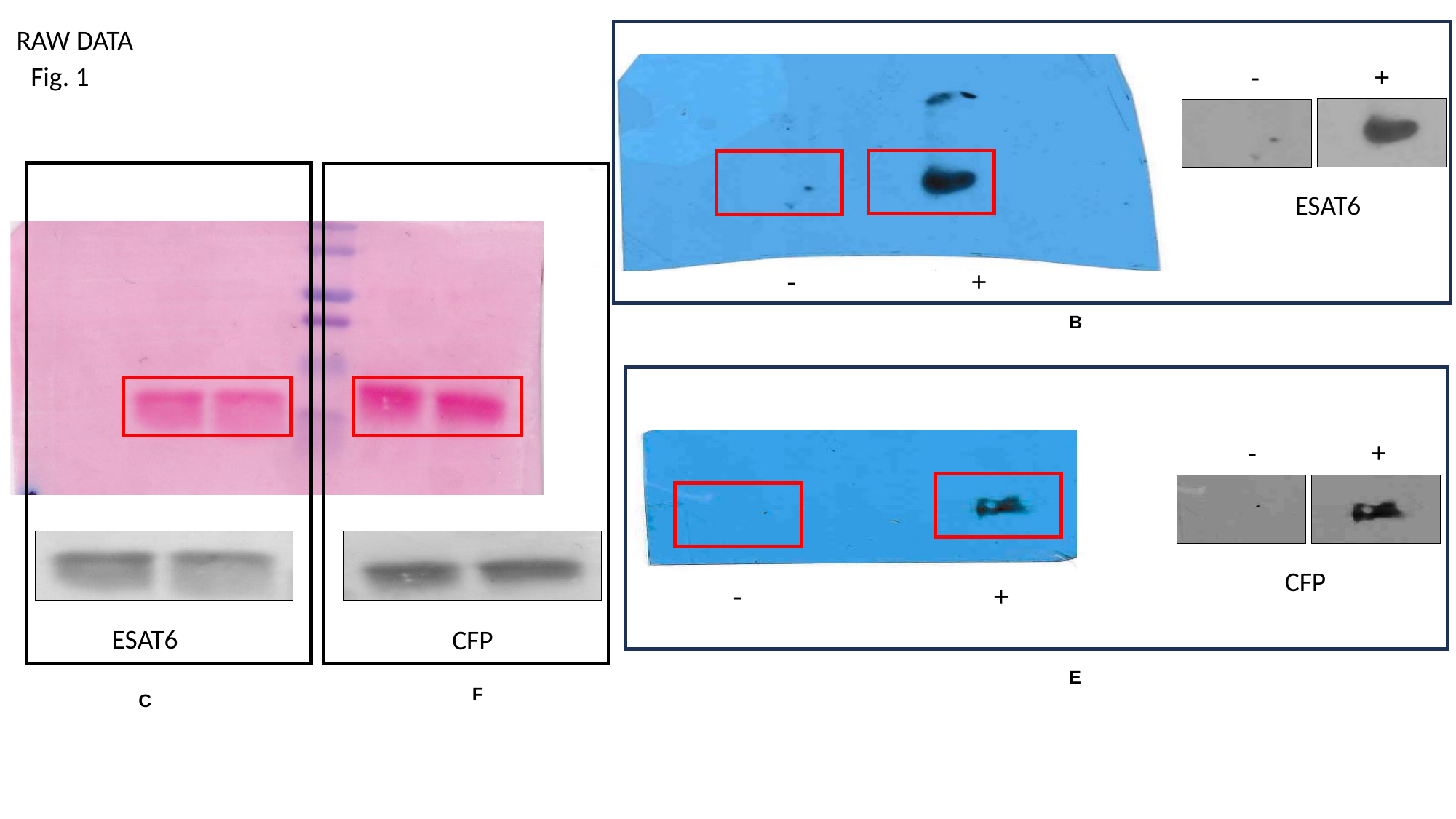

RAW DATA
Fig. 1
 - +
ESAT6
 - +
B
 - +
CFP
 - +
ESAT6
CFP
E
F
C

## Slide 2
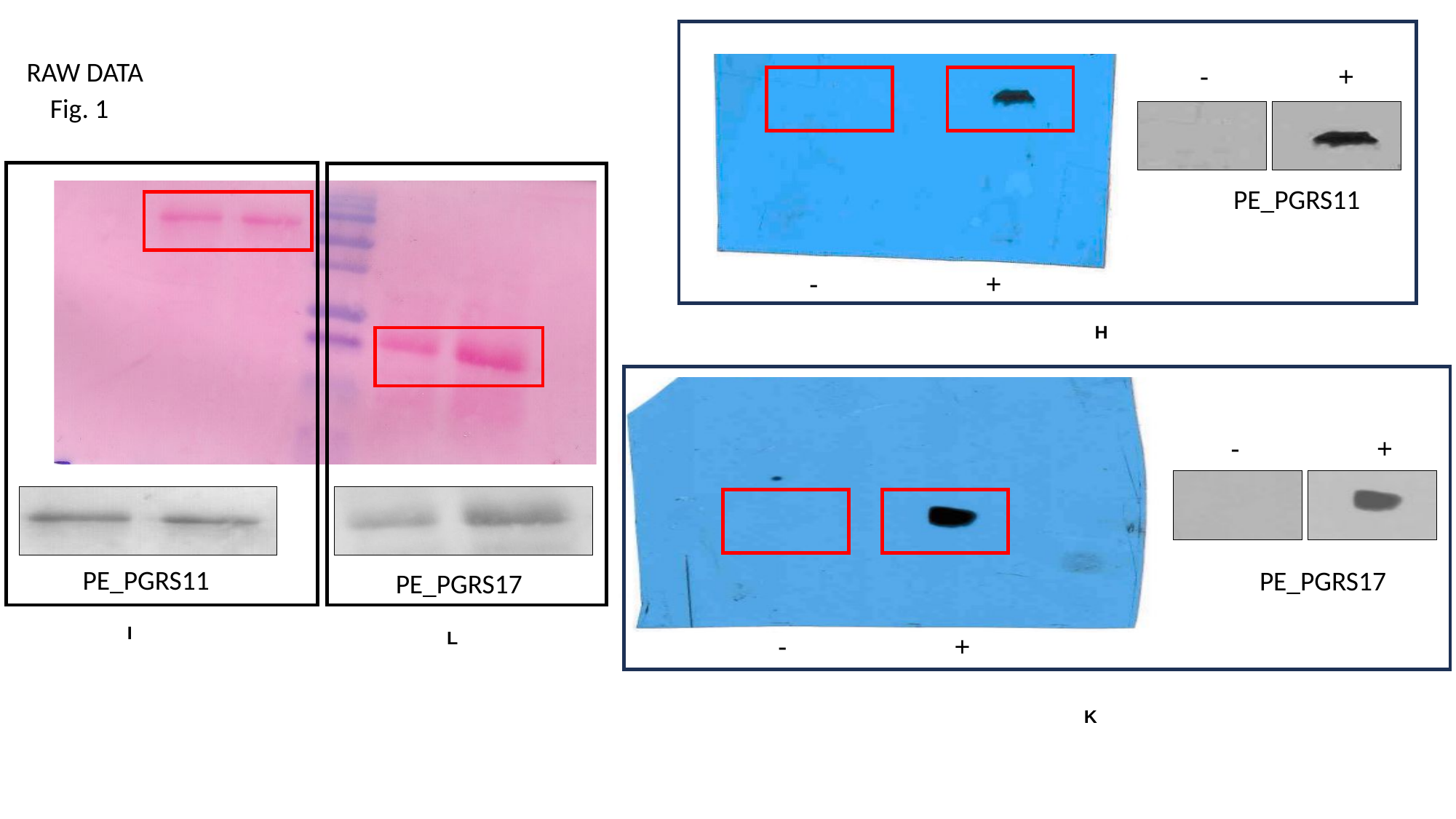

RAW DATA
 - +
Fig. 1
PE_PGRS11
 - +
H
 - +
PE_PGRS11
PE_PGRS17
PE_PGRS17
I
L
 - +
K

Supplement: S1 File — (PPTX) [file pone.0294550.s001.pptx]
